# Environmental Impact Assessment of Using Waste Tires as an Alternative Fuel in a Cement Clinker Production Plant in China: A Case Study

**DOI:** 10.3390/ma19143086

**Published:** 2026-07-17

**Authors:** Wenjuan Li, Jian Wu, Qiongjing Mao, Chengcheng Xu

**Affiliations:** 1 College of Civil Engineering and Architecture, Zhejiang University of Water Resources and Electric Power, Hangzhou 310018, China; maoqj@zuwe.edu.cn (Q.M.); xcc@zuwe.edu.cn (C.X.); 2 Ecological Environment Low Carbon Development Center of Zhejiang Province, Hangzhou 310012, China

**Keywords:** cement clinker, waste tires, alternative fuel, life cycle assessment

## Abstract

Waste tires as an alternative fuel in the cement industry offer multiple advantages, including reduced CO_2_ emissions and decreased reliance on fossil fuels. In this study, a comparative life cycle assessment (LCA) was conducted for cement clinker production with coal (CPC) as fuel and with waste tires as an alternative fuel (CPCT). The study adopted a “gate-to-gate” scope, with a functional unit of 1 ton of clinker. Environmental impacts were evaluated using the IMPACT 2002 + method. Global warming and non-renewable energy were the dominant impacts in cement clinker production. Compared to the CPC scenario, the CPCT scenario reduced the endpoint damage to resources, climate change, and human health by 19.91%, 2.30%, and 0.70%, respectively. However, the damage to ecosystem quality increased by 11.53%. When the waste tires substitution ratio increased from 5% to 20% according to scenario simulation results, the impacts on non-renewable energy and global warming dropped by 16.23% and 8.59%, respectively. Conversely, this higher substitution ratio exacerbated terrestrial acid/nutri (+10.75%), aquatic acidification (+9.70%), and respiratory inorganics (+5.76%). The results indicated a trend toward reduced reliance on coal and lower CO_2_ emissions in the cement clinker production process through the substitution of waste tires. Nevertheless, the trade-off involved higher emissions of certain pollutants, most notably NO_x_, leading to increased ecosystem-related impacts.

## 1. Introduction

China’s cement industry accounts for approximately 57% of the global cement production, and its coal consumption represents about 6% of the national total [1]. Meanwhile, emissions of SO_2_, NO_x_ and particulate matter (PM) from this sector account for approximately 5%, 17%, and 21% of the industrial sector emissions, respectively [2]. Direct CO_2_ emissions account for 12–15% of the total CO_2_ emissions in China [3,4,5,6] and over 60% of the country’s industrial process emissions [7]. Many scholars have identified the cement industry as a key industry for further mitigating air pollutant emissions and achieving China’s “dual carbon” goals [8,9,10].

A vital strategy for cement enterprises to achieve deep reductions in air pollutants and CO_2_ emissions is the full utilization of industrial by-products and wastes as alternative fuels, including refuse-derived fuel (RDF), biomass, waste organic solvents, waste tires, and waste plastics. Among these, waste tires have drawn increasing attention. In 2022, China generated as many as 350 million waste tires, weighing approximately 12.28 million metric tons, yet the resource utilization rate remained below 60% [11]. Most waste tires are discarded in dumps or landfills without proper treatment, posing severe threats to both the environment and human health [12]. Owing to their high and consistent calorific value, waste tires are of interest for recovery in the cement industry. Moreover, the iron contained in waste tires can partially supply the iron normally added to the kiln feed [13].

Cement plants offer excellent conditions for the co-processing of waste tires. In Europe, the waste tires substitution rate in cement manufacturing could reach 5% [14]. Reza et al. suggested that a cement plant with an annual clinker production capacity of 500,000 tons could incorporate 12,000 tons of waste tires [15]. Cankaya reported that a cement plant in India adopted tire-derived fuel as an alternative fuel, achieving a substitution rate of 10% [16]. Wu et al. implemented the retrofitting and application of waste tires as an alternative fuel at a cement enterprise in Yunnan, China, and found that incorporating 3.50% waste tires could reduce the comprehensive standard coal consumption for clinker production to ≤100 kgce t^−1^ clinker [17]. Ye et al. proposed that a tire feed rate of less than 5.0 t h^−1^ could maintain clinker performance while simultaneously reducing coal consumption and lowering SO_2_ and NO_x_ emission concentrations [18]. However, the application of waste tires as an alternative fuel in cement clinker production may pose challenges, including affecting clinker quality, increasing CO_2_ emissions, and causing heavy metal pollution [14]. Therefore, scientific and quantitative assessment methods are essential to validate its environmental benefits.

Currently, life cycle assessment (LCA) is the most widely adopted method for evaluating the environmental impacts of substituting fossil fuels with solid waste in cement clinker production. Fikse et al. conducted a systematic LCA on the substitution of coal with waste tires in US cement kilns, revealing that replacing coal with one ton of waste tires could avoid approximately 543 kg of CO_2_ eq emissions [19]. Kishan et al. employed LCA to demonstrate that waste tires exhibit better environmental performance than coal in terms of climate change, ecosystem quality, human health, and resource depletion [13]. Oscar et al. compared three waste tires disposal scenarios and found that incineration in cement plants exhibited the best environmental performance [20]. Conversely, Georgiopoulou and Lyberatos compared the environmental burdens of using three alternative fuels—biosolids, waste tires, and RDF—concluding that RDF offered superior environmental benefits compared to waste tires [21]. These findings indicated a lack of consensus regarding the environmental impacts of waste tires as an alternative fuel. Guo et al. reviewed international studies and reported that the use of alternative fuels and raw materials could reduce environmental impacts such as global warming potential (GWP), acidification, and freshwater eutrophication by 10–13% [10]. Tang et al. investigated coal gangue as an alternative raw material and noted reduced environmental impact potentials in terms of GWP, human carcinogenic toxicity, and mineral/fossil resource depletion [22]. Based on primary data from a cement plant in Southern Europe, Shang et al. studied a 43% substitution of fossil fuels with RDF and waste tires, reporting reductions of 5%, 14%, 15%, and 17% in non-renewable resource consumption, acidification, eutrophication, and GWP, respectively [23].

The application of waste tires as an alternative fuel in cement clinker production in China is still in its infancy. It was not until the Implementation Plan for Carbon Peaking in the Building Materials Industry, jointly issued by the Ministry of Industry and Information Technology and three other departments of China in 2022, that this practice began to be actively promoted. The policy explicitly calls for “supporting the substitution of coal with combustible wastes” and “focusing on the R&D of fuel substitution technology before 2025” [24]. This policy milestone has catalyzed the emergence of practical cases in cement enterprises. Moreover, existing studies [17,22] have predominantly relied on cement operational data from other countries for their LCA models, resulting in a significant lack of environmental impact assessments specific to the Chinese context. In particular, localized analyses based on actual domestic cement clinker production scenarios remain scarce, meaning the environmental impacts of China’s cement production have not been accurately evaluated.

To address these gaps, this study utilized actual operational data from a cement clinker production plant in China to conduct an LCA case study. It aimed to evaluate the differences in key environmental impacts—including non-renewable resource consumption, acidification, ecotoxicity, human health, and GWP—between waste tires as an alternative fuel and traditional coal-fired cement clinker production. Furthermore, this research identified the critical influencing factors. Ultimately, it not only enriched the current LCA database and case studies but also provided a scientific basis for utilizing waste tires as an alternative fuel to achieve improved environmental performance in the cement industry.

## 2. Materials and Methods

This study was conducted in adherence with standard LCA guidelines (ISO 14040:2006 [25] and ISO 14044:2006 [26]) developed by the International Organization for Standardization. LCA is divided into four main stages according to the ISO 14040 [25]: (1) goal and scope definition; (2) inventory analysis; (3) life cycle impact assessment; and (4) life cycle interpretation [27].

### 2.1. Goal and Scope Definition

**Figure 1:**
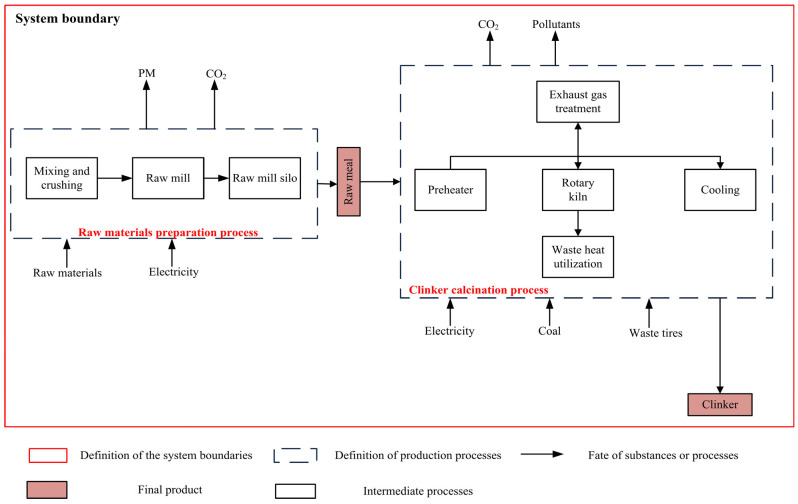
The system boundary of cement clinker production.

A functional unit of 1 ton of cement clinker was defined, based on actual production data from a cement clinker plant in China. The scope of this study was to comparatively analyze the variations in environmental impacts of the cement clinker production process with and without using waste tires as an alternative fuel. In most cement LCA studies, the system boundary follows a “cradle-to-gate” approach, typically encompassing raw material extraction and transportation, cement manufacturing (including raw meal pretreatment, clinker calcination, and cement grinding), and related energy inputs [10]. Constrained by research costs and data availability, this study adopted a “gate-to-gate” approach, and the corresponding system boundary is illustrated in Figure 1.

The “gate-to-gate” approach did not account for the acquisition and transport of waste tires, thus precluding a quantitative assessment of the environmental benefits of waste tires recycling and transportation. Furthermore, it excluded a direct comparison with the environmental impacts associated with coal mining, acquisition, and transport. However, cement clinker production has been a primary focus of regulatory control in China due to its substantial emissions of CO_2_ and pollutants such as PM, NO_x_, and SO_2_. In contrast, the environmental impacts during the raw material extraction and transportation stages were relatively low [28,29]. Therefore, adopting the “gate-to-gate” approach captured the primary emission reduction trends brought by alternative fuels, providing a theoretical basis for both cement enterprises and government management.

### 2.2. Life Cycle Inventory Analysis

Life cycle inventory (LCI) analysis involves the collection, organization, and summarization of input and output data throughout the entire life cycle of cement clinker production. This study utilized actual operational data for the year 2024 obtained from a cement clinker production plant located in Zhejiang Province, China. The plant is a typical medium-to-large-scale producer operating a mainstream new dry-process clinker production line with a daily capacity of 5500 tons. It employs an integrated pollution control system comprising low-NO_x_ combustion technology; selective non-catalytic reduction; selective catalytic reduction; bag filters; and a kiln ash–gypsum desulfurization system to control emissions of PM, SO_2_, NO_x_, NH_3_, and heavy metals. These technologies represent advanced emission control standards in China, reducing the emission concentrations of PM, SO_2_, and NO_x_ below 10 mg/m^3^, 35 mg/m^3^, and 50 mg/m^3^, respectively, which meet the nation’s latest ultra-low emission standards [30].

To enhance energy efficiency, the new dry-process clinker production line is equipped with a 9 MW waste heat power (WHR) generation system. Previous studies have indicated that the electricity saved by such a system accounts for nearly half of the total power consumption in cement clinker production [31]. This enterprise is one of the few cement clinker producers in China that have adopted waste tires as an alternative fuel, demonstrating both technological advantage and representativeness. The LCA results derived from this plant were applicable to other cement clinker producers in China that utilize similar production processes.

The input–output inventory for the coal-fired cement clinker production process using coal (CPC) and the cement clinker production process using waste tires (CPCT) as an alternative fuel are presented in Table 1. Through the on-site investigation, the plant provided comprehensive data for one year, including the consumption of raw materials, coal, and electricity, as well as clinker production and emissions of CO_2_, PM, SO_2_, NO_x_, NH_3_, HCl, HF, and heavy metals, along with the property and substitution ratio of waste tires. All these parameters were ultimately normalized to the functional unit to construct the inventory. As shown in Table 1, using waste tires as an alternative fuel led to increased emissions of only PM and NO_x_, without causing any increase in the emissions of SO_2_, HCl, HF, and heavy metals. The element characteristics of waste tires used in the plant were detailed in Table 2. The mass-based substitution ratio was 5.80%, representing the maximum achievable substitution under current operating conditions of this plant.

### 2.3. Life Cycle Impact Assessment Method

Impact categorization models are generally classified into two types: endpoint models (e.g., Eco-indicator 99, EPS 2000) and midpoint models (e.g., CML 2001, EDIP 2003) [10]. To analyze cement production, it is recommended to adopt a midpoint model or a combined approach utilizing both. For instance, the IMPACT 2002+ method first evaluates intermediate impact subcategories and subsequently aggregates them into the ultimate damage categories [10]. This method was developed as an update integrating results from the IMPACT 2002 model for human health, CML, and Eco-indicator 99. Several scholars have employed the IMPACT 2002+ method to assess the environmental impacts of cement production, demonstrating its feasibility [13,27,32]. Chen et al. [33] further confirmed that the results obtained using the IMPACT 2002+ method were reliable. The midpoint and endpoint environmental impacts, along with their characterization factors, are presented in Table 3.

Normalization and weighting, which were optional stages intended to facilitate the interpretation of results, were not conducted in this study due to the lack of widely recognized reference values for normalization and weighting factors in China. Applying these steps will increase the subjectivity and uncertainty of the evaluation results [34]. This study focused on the changes of environmental impacts resulting from the substitution of waste tires, rather than on the direct comparison of different environmental impact categories within the same scenario. Consequently, the use of the unweighted results could enhance the objectivity of this case study.

The computational process for this case study was conducted using openLCA software 2.4.1. OpenLCA 2.4.1 is an open-source LCA assessment software released by GreenDelta in Germany in 2007. Its primary advantages include low acquisition costs and the ability to import various data formats, such as SimaPro, EcoSpold, and ILCD [35]. Furthermore, it is compatible with China’s independently developed Tiangong database (https://www.tiangong.earth/zh, accessed on 20 May 2023), which greatly facilitates the localization of this case study.

### 2.4. Sensitivity Analysis Method

Sensitivity analysis evaluates the extent to which variations in input data influence the output results, thereby helping to identify the key factors contributing to environmental impacts and providing guidance for the optimization of process conditions [36]. Although Monte Carlo analysis was a popular method in LCA [27], the plant only began using waste tires as an alternative fuel in 2024, making it impossible to perform such an analysis in the absence of extensive historical data. Therefore, in this study, the sensitivity coefficient was employed to characterize the degree of influence of input variables on the environmental impact calculation results, as shown in Equation (1):

(1) $$E_{i}=\frac{{∆A}_{i}/A_{i}}{{∆F}_{i}/F_{i}}$$

where $E_{i}$ is the sensitivity coefficient of the *i* input variable (dimensionless); $A_{i}$ is the environmental impact potential caused by the *i* input variable; ${∆A}_{i}$ is the change in the environmental impact potential caused by the *i* input variable; $F_{i}$ is the value of the *i* variable; ${∆F}_{i}$ is the change in the value of the *i* input variable. A larger $\left|E_{i}\right|$ indicates that the input variable *F_i_* is more sensitive to a specific environmental impact.

## 3. Results and Discussion

### 3.1. Comparison of Environment Impact of CPC and CPCT Scenarios

The midpoint environmental impact scores for CPC and CPCT scenarios are presented in Table 4. Both scenarios exhibited the greatest impact on non-renewable energy, primarily because cement clinker production consumed substantial amounts of non-renewable resources, including limestone, shale, sandstone, and coal [27]. In addition, cement clinker production contributed significantly to GWP, with a potential value reaching 868 kg CO_2_ eq t^−1^ clinker. This indicated that 868 kg CO_2_ eq was emitted for every ton of clinker produced, which was generally consistent with the findings of Cankaya et al. [27], though lower than that reported by Shang et al. [23]. This discrepancy was presumably attributed to the fact that WHR was not considered in the model established by Shang et al. Previous studies have shown that the application of a WHR power generation system can save an average of 24 kWh of electricity per ton of clinker produced [37]. Based on the data published by the International Energy Agency (IEA), which stated that each kWh of electricity generated 0.9746 kg of CO_2_ [38], the adoption of WHR by this enterprise avoided the emission of 23.39 kg CO_2_ eq t^−1^ clinker. Furthermore, the combined GWP reduction from the WHR and CPC scenario approached 906 kg CO_2_ eq t^−1^ clinker in the non-WHR scenario calculated by Shang et al. [23].

The LCA results showed zero scores for aquatic eutrophication, carcinogens, ionizing radiation, land occupation, ozone layer depletion, and respiratory organics, suggesting that cement clinker production did not contribute to these specific environmental impacts. However, significant impacts on ecosystems and human health were observed, specifically in aquatic acidification, aquatic ecotoxicity, non-carcinogenic effects, respiratory inorganics, terrestrial acid/nutri, and terrestrial ecotoxicity. These environmental impacts stemmed primarily from the emissions of PM, SO_2_, NO_x_, NH_3_, HCl, HF, and heavy metals. Our study corroborated the study by Ige and Kabeya [39], which explicitly linked cement clinker production to terrestrial acidification, carcinogenic and non-carcinogenic effects, as well as terrestrial and freshwater ecotoxicity. The consistency underscores the reliability of the identified environmental impacts.

**Figure 2:**
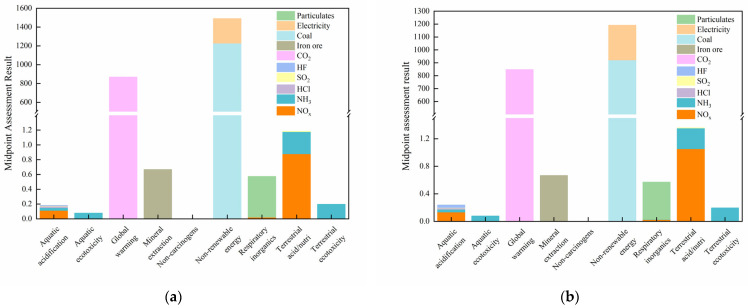
Major substance contributions to midpoint impact: (**a**) CPC and (**b**) CPCT.

The contributions of major substances to the environmental impacts of CPC and CPCT scenarios are given in Figure 2. In the CPC scenario, the high level of non-renewable energy was related to coal and electricity, which accounted for 81.18% and 18.39%, respectively. CO_2_ was the sole contributor to GWP, similar to the CPC scenario. Major contributors to terrestrial acid/nutri were NO_x_ and NH_3_, responsible for 74.16% and 25.27%, respectively. In the CPCT scenario, coal and electricity remained the dominant contributors to non-renewable energy, accounting for 77.18% and 22.65%, respectively; the reduced share of coal reflects the partial substitution by waste tires. A high level of terrestrial acid/nutri was still related to NO_x_ and NH_3_, whose contributions were 80.80% and 22.94%, respectively.

Contrary to previous studies suggested that using waste tires as an alternative fuel increases heavy metal emissions and exacerbates the ecological and human toxicity impacts of the clinker production process [40,41], actual operational data provided from the cement plant (Table 1) demonstrated that the addition of waste tires did not increase the total heavy metal emissions. Notably, Fe and Zn present in waste tires (in Table 2) did not volatilize into the exhaust stream. Narra et al. experimentally verified that waste tires as an alternative fuel do not increase the concentrations of heavy metals in the exhaust gas of a cement kiln, when the cement plant has sufficient fly ash capture capacity [42]. Arfala et al. further elucidated that waste tires exhibited the lowest heavy metal contents among alternative fuels, with elevated emission concentrations of Cr, Ni, and Zn primarily attributable to coalmine wastes and petroleum coke [43]. As illustrated in Figure 2, heavy metals contributed negligibly to the total environmental impacts. Consistent with these findings, Ige and Kabeya also found that adding alternative fuels could reduce the introduction of certain metals, such as mercury, into the clinker production system [39].

Compared with the CPC scenario, the CPCT scenario showed a 19.92% reduction in non-renewable energy impact and a 2.30% reduction in GWP, owing to lower coal consumption and CO_2_ emissions. Conversely, the impacts of terrestrial acid/nutri, aquatic acidification and respiratory inorganics increased by 10.15%, 7.69% and 1.23%, respectively. These increases were driven by higher NO_x_ emissions resulting from the use of waste tires as an alternative fuel, as NO_x_ was the dominant contributor to these impact categories. The remaining environmental impact categories showed no change.

**Figure 3:**
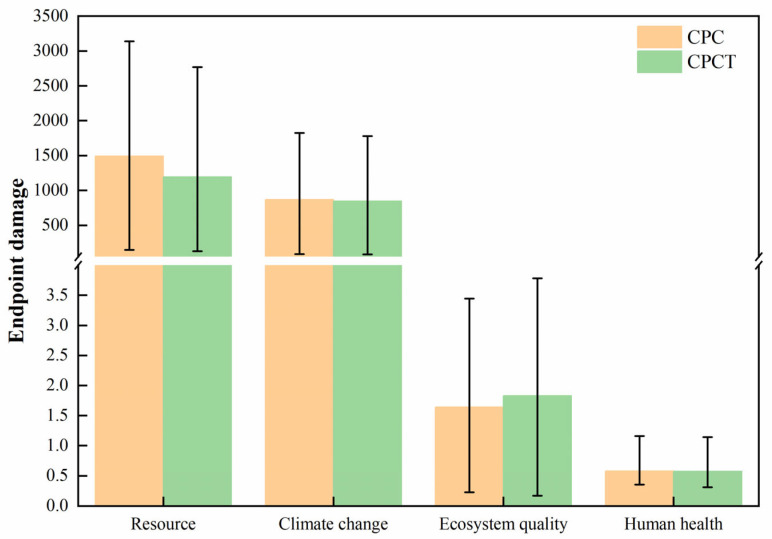
Endpoint damage assessment scores in CPC and CPCT.

Based on the IMPACT 2002+ method, the midpoint environmental impacts were aggregated into endpoint damage categories, as illustrated in Figure 3. The damage to resource, climate change, ecosystem quality and human health in CPC was 1490.28 (1341.25–1648.37) MJ primary, 868 (781.20–954.80) kg CO_2_ eq, 1.64 (1.41–1.80) PDF·m^2^·yr, 0.58 (0.22–0.59) DALY. The damage to resources, climate change, ecosystem quality and human health in CPCT was 1193.62 (1065.04–1573.96) MJ primary, 848 (763.20–932.80) kg CO_2_ eq, 1.83 (1.66–1.95) PDF·m^2^·yr, 0.57 (0.26–0.57) DALY. Compared to the CPC scenario, the CPCT scenario achieved reductions in damage to resources, climate change and human health of 19.91%, 2.30% and 0.70%, respectively. However, the damage to ecosystem quality increased by 11.53%. Thus, while utilizing waste tires as an alternative fuel offered significant advantages in mitigating climate change and conserving resources, its potential adverse effects on ecosystem quality warrant careful attention.

### 3.2. Contribution Analysis of Environmental Impacts by Production Processes

**Figure 4:**
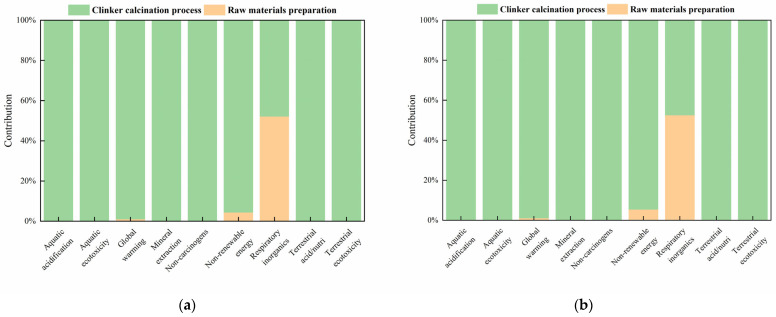
Contributions of each process to the midpoint environmental impacts: (**a**) CPC and (**b**) CPCT.

This study further analyzed the contributions of the raw material preparation and clinker calcination processes to the midpoint environmental impacts, as shown in Figure 4. In both CPC and CPCT scenarios, the clinker calcination process accounted for the entirety of aquatic acidification, aquatic ecotoxicity, mineral extraction non-carcinogens, terrestrial acid/nutri, terrestrial ecotoxicity (100% each), as well as 98.87% of global warming, and 94.53% of non-renewable energy impacts. These findings indicated that the clinker calcination process dominated the overall midpoint environmental impacts of the cement clinker production system.

The raw materials preparation process was the dominant contributor to respiratory inorganics, mainly due to PM emissions from this process. Cankaya et al. [27] reported that raw material preparation contributed less to respiratory inorganics than the clinker calcination process, contrary to the finding of this study. This discrepancy was presumably attributed to the higher level of PM emitted during raw material preparation at the investigated plant compared with the clinker calcination stage. A proportion of 4.38% in non-renewable energy and 1.11% in GWP was attributed to the raw material preparation process, indicating that the energy consumption and CO_2_ emission from this process represent only a small fraction of the total cement clinker production.

**Figure 5:**
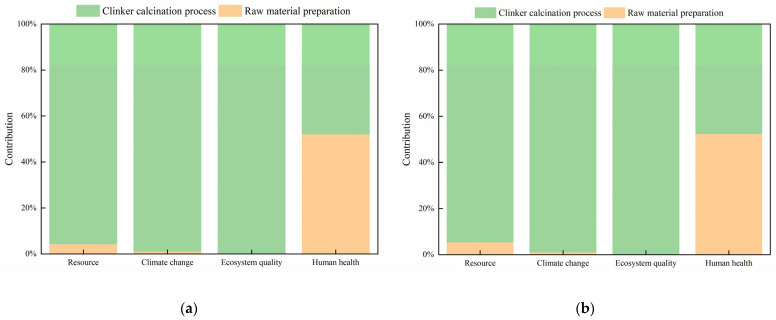
Contributions of each process to the endpoint damage: (**a**) CPC and (**b**) CPCT.

In the endpoint damage analysis (Figure 5), the clinker calcination process contributed 100% to ecosystem quality, 98.89% to climate change, 95.63% to resources, and 47.92% to human health. The raw material preparation stage was responsible for 52.07% of human health, 4.38% of resources, and 1.11% of climate change impacts. Those contribution ratios were consistent with the results of the midpoint environmental impacts.

### 3.3. Impact Analysis of Different Substitution Ratios of Waste Tires

**Figure 6:**
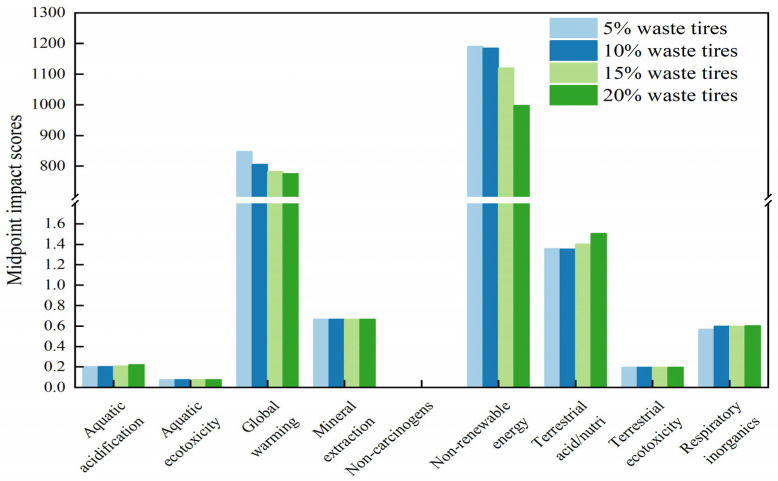
Changes in midpoint environmental impacts with waste tires substitution ratios ranging from 5% to 20%.

Given that the waste tires substitution ratio at the investigated cement clinker production plant was only 5.80%, this single data point was insufficient to reveal the effect of varying substitution ratios on the environmental impacts. To address this problem, four additional scenarios were established with the substitution rates of waste tires of 5%, 10%, 15%, and 20%. By adjusting the input and output parameters in openLCA 2.4.1, the corresponding changes in environmental impacts were calculated, as shown in Figure 6. Although this section relied on scenario simulation and predicted data, it could reflect the trends of environmental impact variations associated with increasing substitution rates.

A higher waste tires substitution ratio led to a lower score for non-renewable energy, primarily due to the reduction in coal consumption. As the substitution ratio increased from 5% to 20%, non-renewable energy and GWP decreased by 16.23% and 8.59%, respectively. This indicated that replacing a greater proportion of coal with waste tires mitigated both non-renewable energy and GWP impacts.

Conversely, terrestrial acid/nutri, aquatic acidification, and respiratory inorganics increased by 10.75%, 9.70%, and 5.76%, respectively, as the substitution ratio increased. This was mainly because higher waste tires utilization led to elevated emissions of SO_2_, NO_x_, and PM. Ige and Kabeya [39] found that increasing the alternative fuel substitution rate up to 20% resulted in increased SO_2_ and NO_x_ emissions through LCA. Similarly, Vasiliu et al. experimentally demonstrated that adding 10% waste tires raised the emission concentrations of PM, SO_2_, and NO_x_ [14], which aligned with the scenario predictions of this study.

The increased use of waste tires had no significant impact on aquatic ecotoxicity, mineral extraction, non-carcinogens, and terrestrial ecotoxicity. Mineral extraction was attributed to iron ore consumption, while aquatic ecotoxicity, terrestrial ecotoxicity, and non-carcinogens were mainly driven by NH_3_ emission. This indicated that both iron ore consumption and NH_3_ emission were essentially independent of waste tires utilization. It further suggested that waste tires could not serve as an iron source for cement clinker production.

In terms of endpoint damages, as the substitution ratio increased from 5% to 20%, the damages to climate change and resources decreased by 8.59% and 8.12%, respectively, whereas ecosystem quality and human health impacts increased by 5.11% and 2.88%, driven by rising NO_x_ emission. These endpoint trends were consistent with the observed change in midpoint environmental impacts. Therefore, stricter NO_x_ emission control measures were necessary to alleviate damage to ecology and human health as the substitution ratio increases.

### 3.4. Sensitivity Analysis

**Figure 7:**
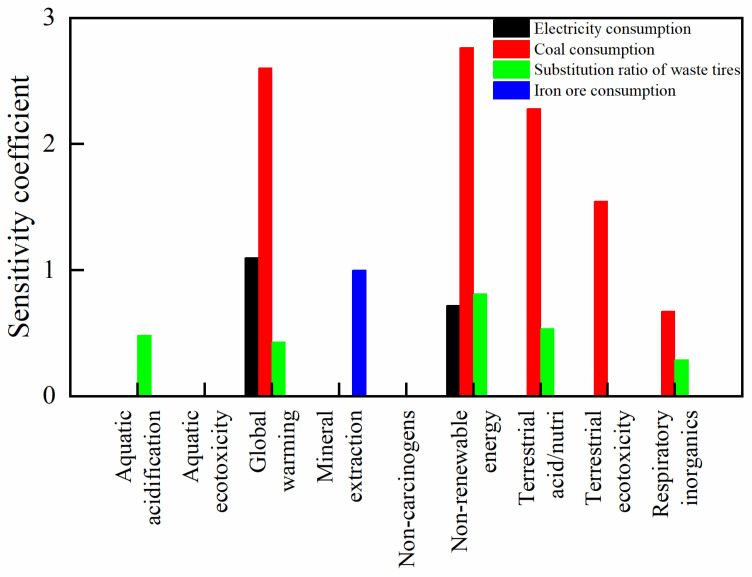
The sensitivity coefficients of different inputs to midpoint environmental impact.

Based on Figure 2, electricity consumption, coal consumption, and iron ore consumption were identified as the dominant contributing inputs. Notably, coal consumption is a primary source of pollutants such as PM, SO_2_, and NO_x_ during clinker production; thus, variation in coal consumption indirectly affected the associated environmental impacts. Moreover, the waste tires substitution ratio directly influenced the consumption of both coal and waste tires, making it another critical input variable. Hence, electricity consumption, coal consumption, waste tires substitution ratio, and iron ore consumption were selected as the four input variables contributing most significantly to the midpoint environmental impacts. Using Equation (1), the sensitivity coefficients of these four inputs were calculated, with the results presented in Figure 7. In the calculation, these four variables were assumed to vary within a ±10% range, while all other inventory parameters were held constant.

Iron ore consumption influenced only mineral extraction, with a sensitivity coefficient of 1, indicating a directly proportional relationship. Electricity consumption was sensitive to non-renewable energy and GWP; its sensitivity coefficient for GWP exceeded 1, indicating that electricity was a sensitive factor for this category. Coal consumption exhibited the highest sensitivity to GWP, non-renewable energy, terrestrial acid/nutri, and terrestrial ecotoxicity, with all sensitivity coefficients exceeding 1. This confirmed coal consumption as a sensitive factor for these four impact categories, primarily because coal combustion in cement kilns not only generated CO_2_ but also served as the main source of SO_2_, NO_x_, and PM emissions. The waste tires substitution ratio showed sensitivity to aquatic acidification, GWP, non-renewable energy, terrestrial acid/nutri, and respiratory inorganics impacts; however, none of its coefficients exceeded 1, mainly due to the relatively low mass substitution of waste tires.

## 4. Conclusions

Using actual operational data from a cement clinker plant in China, this study comparatively assessed the life cycle environmental impacts of two clinker production scenarios: conventional coal-fired production (CPC) and production with waste tires as an alternative fuel (CPCT). Global warming and non-renewable energy were identified as the dominant impact categories. A high level of non-renewable energy was associated with coal and electricity, while global warming had a sole contributor: CO_2_ emissions.

At a 5.80% substitution ratio of waste tires, the CPCT scenario reduced the non-renewable energy and global warming by 19.92% and 2.30%, respectively, relative to the CPC scenario. Scenario simulations with substitution ratios ranging from 5% to 20% revealed that non-renewable energy and global warming decreased by 16.23% and 8.59%, indicating that replacing more coal with waste tires mitigates both impacts. These findings preliminarily suggested that utilizing waste tires as an alternative fuel could further reduce cement clinker producers’ reliance on coal and contribute to CO_2_ emission reduction. The sensitivity analysis identified electricity and coal consumption as the two most critical factors driving the environmental impacts of cement clinker production, with all sensitivity coefficients exceeding 1, confirming their high sensitivity.

These results could be useful for waste managers at cement plants and decision makers in China. However, the gate-to-gate approach in this study excluded the collection and transportation of waste tires, which may affect the overall life cycle environmental impacts. Moreover, the assessment was limited to environmental aspects without considering economic aspects. In the future, a comprehensive “cradle-to-grave” evaluation should be conducted, incorporating the environmental impacts of waste tire recycling and transportation, and integrating economic analysis through more extensive data collection. Once sufficient data become available, comprehensive uncertainty analysis and sensitivity analysis will be essential for in-depth investigation. Additionally, since trace pollutant emissions from waste tires were not found to be significant in this study, future research should explore the potential environmental impacts of trace pollutants from waste tires of different sources and compositions.

## Figures and Tables

**Table 1:** The LCI of CPC and CPCT.

Type	Substance	Unit	CPCT	CPC
Input	Limestone	t t^−1^ clinker	1.249	1.249
	Shale	t t^−1^ clinker	0.121	0.121
	Sand stone	t t^−1^ clinker	0.17	0.17
	Iron ore	t t^−1^ clinker	0.023	0.023
	Gypsum	t t^−1^ clinker	0.073	0.073
	Coal	t t^−1^ clinker	0.118	0.124
	Electricity	kWh t^−1^ clinker	72.780	75.070
	Waste tires	t t^−1^ clinker	0.0062	-
Output	Clinker	t t^−1^ clinker	1	1
	CO_2_	t t^−1^ clinker	0.848	0.868
	PM	t t^−1^ clinker	11	10
	SO_2_	g t^−1^ clinker	6	7
	NO_x_	g t^−1^ clinker	182	160
	NH_3_	g t^−1^ clinker	20	20
	HCl	g t^−1^ clinker	24	24
	HF	g t^−1^ clinker	2.4	2.4
	Heavy metals (Be + Cr + Sn + Sb + Cu + Co + Mn + Ni + V)	g t^−1^ clinker	0.83	0.83

**Table 2:** The elemental analysis of waste tires (%).

C	H	O	N	F	S	Fe	Zn	Cl	LHV (MJ kg^−1^)
69.00	6.20	3.50	0.35	0.50	0.80	11.50	1.40	0.04	21.76

**Table 3:** Environmental impact categories and their units in IMPACT 2002+ method.

Midpoint Category	Equivalent Unit	Endpoint Category
Non-renewable energy	MJ primary resource	Resource
Mineral extracting	MJ surplus	
Global warming	kg CO_2_ eq	Climate Change
Ozone layer depletion	kg CFC-11 eq	Ecosystem quality
Aquatic ecotoxicity	kg TEG water	
Terrestrial ecotoxicity	kg TEG soil	
Aquatic acidification	kg SO_2_ eq	
Terrestrial acid/nutri	kg SO_2_ eq	
Land occupation	m^2^	
Aquatic eutrophication	kg PO_4_ P-lim	
Respiratory inorganics	kg PM_2.5_ eq	Human health
Carcinogens	kg C_2_H_3_Cl eq	
Ionizing radiation	Bq C-14 eq	
Non-carcinogens	kg C_2_H_3_Cl eq	
Respiratory organics	kg C_2_H_4_ eq	

**Table 4:** Comparison of environmental impact of CPC and CPCT.

Midpoint Impact	CPC	CPCT
Aquatic acidification	0.182 (0.160–0.200)	0.196 (0.183–0.216)
Aquatic ecotoxicity	0.078 (0.062–0.086)	0.078 (0.070–0.086)
Aquatic eutrophication	0	0
Carcinogens	0	0
Global warming	868 (781.2–954.8)	848 (763.2–932.8)
Ionizing radiation	0	0
Land occupation	0	0
Mineral extraction	0.667 (0.600–0.734)	0.667 (0.600–0.734)
Non-carcinogens	0.001 (0.0008–0.0011)	0.001 (0.0009–0.0011)
Non-renewable energy	1489.608 (1340.647–1647.637)	1192.952 (1064.437–1573.228)
Ozone layer depletion	0	0
Respiratory inorganics	0.575 (0.222–0.585)	0.571 (0.262–0.575)
Respiratory organics	0	0
Terrestrial acid/nutri	1.184 (1.036–1.303)	1.304 (1.223–1.434)
Terrestrial ecotoxicity	0.196 (0.157–0.216)	0.196 (0.176–0.216)

## Data Availability

The original contributions presented in this study are included in the article. Further inquiries can be directed to the corresponding author.

## Abbreviations

The following abbreviations are used in this manuscript:

LCA	Life cycle assessment.
PM	Particulate matter.
GWP	Global warming potential.
CPC	Clinker production with coal.
CPCT	Clinker production with waste tires.
LHV	Lower heating value.
RDF	Refuse-derived fuel.
WHR	Waste heat recovery.
